# Low cadence interval training at moderate intensity does not improve cycling performance in highly trained veteran cyclists

**DOI:** 10.3389/fphys.2014.00034

**Published:** 2014-01-31

**Authors:** Morten Kristoffersen, Hilde Gundersen, Stig Leirdal, Vegard V. Iversen

**Affiliations:** ^1^Department of Sports and Physical Education, Bergen University CollegeBergen, Norway; ^2^Department of Sports, Nesna University CollegeNesna, Norway

**Keywords:** interval training, low-cadence, efficiency, cycling performance, endurance

## Abstract

**Purpose:** The aim of the present study was to investigate effects of low cadence training at moderate intensity on aerobic capacity, cycling performance, gross efficiency, freely chosen cadence, and leg strength in veteran cyclists.

**Method:** Twenty-two well trained veteran cyclists [age: 47 ± 6 years, maximal oxygen consumption (VO_2max_): 57.9 ± 3.7 ml · kg^−1^ · min^−1^] were randomized into two groups, a low cadence training group and a freely chose cadence training group. Respiratory variables, power output, cadence and leg strength were tested before and after a 12 weeks training intervention period. The low cadence training group performed 12 weeks of moderate [73–82% of maximal heart rate (HR_max_)] interval training (5 × 6 min) with a cadence of 40 revolutions per min (rpm) two times a week, in addition to their usual training. The freely chosen cadence group added 90 min of training at freely chosen cadence at moderate intensity.

**Results**: No significant effects of the low cadence training on aerobic capacity, cycling performance, power output, cadence, gross efficiency, or leg strength was found. The freely chosen cadence group significantly improved both VO_2max_ (58.9 ± 2.4 vs. 62.2 ± 3.2 ml · kg^−1^ · min^−1^), VO_2_ consumption at lactate threshold (49.4 ± 3.8 vs. 51.8 ± 3.5 ml · kg^−1^ · min^−1^) and during the 30 min performance test (52.8 ± 3.0 vs. 54.7 ± 3.5 ml · kg^−1^ · min^−1^), and power output at lactate threshold (284 ± 47 vs. 294 ± 48 W) and during the 30 min performance test (284 ± 42 vs. 297 ± 50 W). Moreover, a significant difference was seen when comparing the change in freely chosen cadence from pre- to post between the groups during the 30 min performance test (2.4 ± 5.0 vs. −2.7 ± 6.2).

**Conclusion**: Twelve weeks of low cadence (40 rpm) interval training at moderate intensity (73–82% of HR_max_) twice a week does not improve aerobic capacity, cycling performance or leg strength in highly trained veteran cyclists. However, adding training at same intensity (% of HR_max_) and duration (90 min weekly) at freely chosen cadence seems beneficial for performance and physiological adaptations.

## Introduction

Success in competitive cycling is dependent on high muscular and aerobic power as well as an effective application of the power to the crank system. According to the training principle of specificity, most cyclists perform the majority of their training on the cycle, including different types of training that promotes specific muscular strength and endurance, as well as technique and efficiency.

Previous studies indicate that muscular strength and endurance (Ettema and Loras, 2009; Leirdal and Ettema, 2009) as well as pedaling technique (Zameziati et al., 2006; Korff et al., 2007; Leirdal and Ettema, 2011) are related to gross efficiency, and that strength training improves cycling performance (Sunde et al., 2010; Ronnestad et al., 2011). Moreover, strength training is shown to increase efficiency in the working muscle (Barrett-O'Keefe et al., 2012). One hypothesis is that muscular strength improvement creates a decrease in the fraction of maximal pedal force necessary for each pedal thrust, thereby shifting the pattern of muscle fiber recruitment toward type I fibers, resulting in reduced energy expenditure (Hickson et al., 1988).

The pedaling rate each min (cadence) is also related to cycling performance, and studies have shown that muscular strength reduces freely chosen cadence (Hansen et al., 2007). Several studies have examined the effects of cadence and its effects on gross efficiency and cycling performance. Most studies have demonstrated a reduction in gross efficiency with increased cadence (Samozino et al., 2006; Leirdal and Ettema, 2009), and that the optimal cadence for best gross efficiency (Foss and Hallen, 2004; Lucia et al., 2004) and performance (Foss and Hallen, 2005) is below the freely chosen cadence for most cyclist. Foss and Hallen (2005) found that a cadence of 80 revolutions per min (rpm) was optimal for cycling performance compared to a cadence of 60-, 100-, and 120 rpm in elite cyclists (24 ± 3 years). Best cycling performance was however seen at freely chosen cadence of 90 rpm (range 80–100). Lucia et al. (2001) found in another study a cadence of 90 rpm (89.3 ± 1.0 and 92.4 ± 1.3) to be optimal in professional cyclists.

Nimmerichter et al. (2012) have previously conducted a study evaluating effects of low cadence training. They found that low cadence (60 rpm) up-hill interval training increased the work rate by 4.4% in a 20 min uphill time-trial and by 1.5% in a 20 min flat time trial. Nimmerichter et al. (2012) suggests the higher forces produced during the low cadence intervals to be beneficial to improve cycling performance. However, caution is required when interpreting the results as motor recruitment patterns differ between cycling uphill vs. flat. Another study performed by Paton et al. (2009) also found low cadence interval training (60–70 rpm) to improve performance in well-trained cyclists, suggesting increased pedal force to be a possible explanation along with an increase in testosterone level. Whether a further reduction in cadence, and thereby increased force production, would improve cycling efficiency and performance to a greater extent needs further examination.

Low cadence training at 40 rpm at moderate intensity [73–82% of maximal heart rate (HR_max_)] is a common training method in Norway both among elite cyclists (personal communication with elite cycling coaches in Norway) and among veteran cyclists. Effects of this training method have, to our knowledge, not been investigated thoroughly.

Cycling has become very popular the last decade in Norway, and participation in cycling races among veterans has exploded. Many veteran cyclists perform at a high level, practicing many hours weekly, and are seeking training methods for best performance.

Therefore, the aim of the present study was to investigate effects of low cadence interval training performed at 40 rpm with an intensity of 73–82% of HR_max_ on cycling performance [30 min cycling performance test, maximal oxygen consumption (VO_2max_), lactate threshold, freely chosen cadence, gross efficiency, and leg strength] in veteran cyclists. We hypothesized that the higher muscular training loads during low cadence training would be beneficial for cycling performance.

## Materials and methods

All tests were performed in the physiological testing laboratory at Bergen University College. The study was approved by the Norwegian Social Science Data Services (NSD), and all participants signed an informed consent before entering the study.

### Participants

Twenty-two well trained male veteran cyclists (age: 47 ± 6 years, body weight: 78 ± 7 kg, VO_2max_: 57.9 ± 3.7 ml · kg^−1^ · min^−1^), who all had completed a Norwegian bicycle race (Bergen-Voss: 165 km including 2100 m climbing) faster than 4 h and 30 min (average speed of 37 km · h^−1^), were invited and included in the study. The study was performed post season, and none of the participants had performed low cadence interval training in a period of 2 months prior the study.

### Experimental design

The participants were randomly assigned into two groups: a low cadence group (*n* = 11, 47 ± 7 years) and a freely chosen cadence group (*n* = 11, 47 ± 5 years). A randomized controlled trial study design was used, with a 12 weeks training intervention period between pre- and posttests. Pre- and posttests were performed on 3 different days separated by 1 day of rest. Lactate threshold and VO_2max_ were tested on the first testing day, the 30 min performance test on the second testing day, whereas leg strength tests were performed on the third testing day. The testing order was the same for all participants, and for pre- and posttests. To avoid learning effect all participants had one familiarization session on the ergometer and in the strength testing apparatus 1 week prior the pretests.

### Training procedures

During the 12 weeks between the pre- and posttests, the low cadence group performed interval training as group sessions on spinning bikes (Abilica, Mylna AS, Norway) two times a week, in addition to their usual training. All participants were thoroughly instructed by a professional cycling coach on how to perform the low cadence interval training [5 × 6 min at a HR of 73–82% of HR_max_ measured at the VO_2max_ test prior the intervention period, with 3 min active rest at freely chosen cadence and low intensity (60–72% of HR_max_) in-between]. Each low cadence session commenced with a 15 min warm up at freely chosen cadence and low intensity. In total, the low cadence group added 60 min of low cadence interval training (73–82% of HR_max_), 30 min at freely chosen cadence at moderate intensity (60–72% of HR_max_) (i.e., the active rest in-between the intervals), and 50 min at freely chosen cadence at low intensity (i.e., the warm up and cool down) per week.

**Figure 1:**
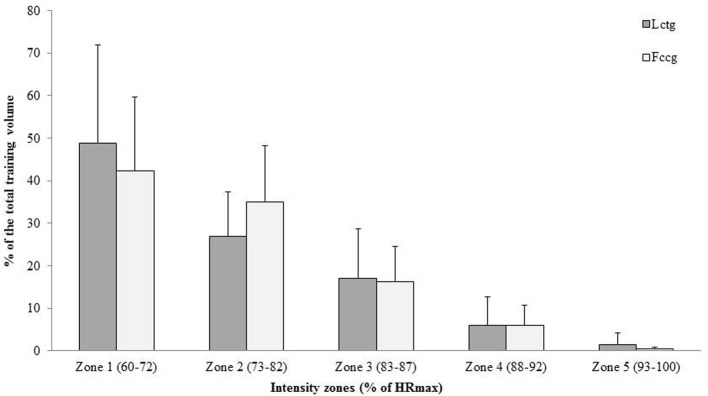
**Endurance training (mean ± *SD*) in % of total training volume at different intensitie zones in the low cadence training group (Lctg) and in the freely chosen cadence group (Fccg) during the 12 weeks training intervention period**. There was no significant differences between the groups.

The freely chosen cadence group added 90 min of cycling at freely chosen cadence at moderate intensity (73–82% of HR_max_) to their usual training. Total training volume and intensity during the intervention period were monitored by recordings from HR monitors (Polar, Kempele Finland), in addition to submission of personal training diaries (mean HR and duration of each training session) (Figure 1). Totally, participants in the low cadence training group (*n* = 11) performed 91 ± 31 h of training during the 12 weeks, while participants in the freely chosen cadence group (*n* = 10) did 88 ± 34 h of training during the same period.

### Testing procedures

Participants were instructed to have no strenuous exercise the day before testing. They were also told not to eat, and to consume products containing caffeine 3 h prior the tests. Pre- and posttests were performed at the same time of the day to exclude possible diurnal effects. All tests on the ergometer were performed under similar environmental conditions (18–20°C).

The cycling tests were performed on the same ergometer (Velotron, Racermate Inc., Washington, USA), with a computer controlled electromagnetic brake mechanism. The Velotron generates a constant power independent of cadence. The participants wore their own cycling shoes, and the cycle was adjusted to their preferred sitting position (same position for the pre- and posttests). Pedal rate each min (cadence) was recorded using the ergometers recording system, and stored on a pc. Freely chosen cadence, which were blinded for the participants, was used at all tests.

Gas exchange values were measured by using an Oxycon Pro apparatus (Jaeger GmbH, Hoechberg, Germany) with mixing chamber. The flow meter was calibrated with a 3 L volume syringe (Hans Rudolph Inc., Kansas City, MO). Before each measurement, the volume of oxygen (VO_2_) and volume of carbon dioxide (VCO_2_) gas analyzers were calibrated using high-precision gases (16.00 ± 0.04% O_2_ and 5.00 ± 0.1% CO_2_, Riessner-Gase GmbH & co, Lichtenfels, Germany). HR was measured with a HR monitor (Polar S610, Polar Electro OY, Kempele, Finland), using a 5-s interval for data storage. VO_2max_ was defined as the highest 1-min average VO_2_ during the test. Maximal HR was defined as the highest value that was attained, in average over a 5 s period at the final stage of the protocol. VO_2_ consumption, respiratory exchange ratio (RER), volume of expired air (VE), power output, measured in watt (W), and HR were recorded and stored during the cycling tests, whereas blood lactate concentration was measured by taking 5 μL samples from the fingertip by a Lactate Scout (SensLab GmbH, Germany).

### Lactate threshold and maximal oxygen consumption

On the first testing day, after a warm-up of 10 min at cycling loads between 100–150 W each participant performed a lactate threshold test starting with a load of 150 W, increasing with 25 W every 5th min. Onset of blood lactate accumulation (OBLA) was defined as a blood lactate concentration of 4 mmol/l hemolysed blood was exceeded. Oxygen consumption was averaged during 3.5–4.5 min period of each stage, and blood sample was taken during the last 15 s at each stage. After reaching OBLA of 4 mmol/l participants rested for three min, before an incremental test to voluntarily exhaustion for determination of maximal oxygen consumption (VO_2max_) was performed. The test started at one load below OBLA (4 mmol/L), and increased with 25 W every 1 min until exhaustion. Blood samples were taken within 1 min after the test for lactate measurement. Exhaustion was defined as meeting three of the following four criteria's (Bassett and Howley, 2000):

1. HR within 5 beats from the participants self-reported HR_max_
2. Lactate level above 8.0 mmol/l hemolyzed blood
3. RER > 1.15
4. VO_2_ consumption which stops increasing or starts decreasing with increased work rate

VO_2max_ and peak power output are average values obtained during the final min of the test.

### 30 min performance test

On the second test day, after a warm-up of 20 min at cycling loads between 100–150 W participants performed a 30 min performance test at freely chosen cadence and load. VO_2_ consumption was measured between 8–10, 18–20, and 28–30 min. Blood lactate concentrations was measured at 10, 20, and 30 min of cycling. HR, power output and rpm was measured throughout the entire test. Performance was measured as mean power output (total watt divided by time in seconds).

### Strength tests

On the third testing day maximal leg strength were tested after a 10 min warm up on a spinning bike (Abilica, Mylna AS, Norway). Participants were tested in two modes; one leg extension, and one leg press (Cybex, Cybex international, USA). A standard protocol to obtain 1repitition maximum (RM) was used. The protocol consisted of three sets with a gradually increasing load (50, 70, and 90% of predicted 1RM from the familiarization test) and a decreasing number of repetitions (8, 5, and 4). The first 1RM attempt was performed with a load 2.5 kg below predicted 1RM. The load was increased by 2.5–5 kg until the cyclist failed to lift the same load after two attempts. A three min rest was given between each attempt.

### Gross efficiency

Gross efficiency was defined as the power divided by the metabolic rate (Ettema and Loras, 2009). The aerobic energetic metabolic rate was determined form VO_2_ and VCO_2_ by calculating the product of VO_2_ and the oxygen energetic equivalent by using the associated measurements of RER, and standard conversion tables for the energetic equivalent for oxygen consumption. The net anaerobic metabolic rate was calculated from the obtained blood lactate values measured directly after 5 min at each load. A 1 mmol/l increase of blood lactate per min was regarded as equivalent to 3 ml/kg of oxygen consumed with an RER = 1 (di Prampero and Ferretti, 1999). Gross efficiency in the lactate threshold test was calculated from three last loads before reaching OBLA and in the performance test as the ratio between the average work rate and the average metabolic rate through the test.

### Statistics

The Statistical Products of Service Solution package (SPSS Statistics, version 21) was used for all statistical analyses. Descriptive data are shown as mean ± standard deviation (*SD*). Skewness and Kurtosis were used to test normality for all data. Paired-samples *t*-tests were used for within-subject analyses in both groups for all variables. Independent-samples *t*-tests were used for comparisons between the two groups before and after the training intervention, and also when comparing changes from pre- to posttest between groups. A *p*-value of = 0.05 was considered as statistically significant. The magnitude of changes was expressed as standardized mean differences (effect size, ES) (Cohen, 1988). *ES*-values between 0.2–0.49 indicated a small difference, between 0.5–0.79 as a medium difference, and from 0.8 and above as a large difference.

## Results

There were no significant differences between the two groups in any variable at the pretest. Also, through the training period, there was no significant difference between the groups in training load (intensity and time) (Figure 1). Also, there was no significant change in body weight in either of the two groups from pre- to posttests.

### VO_2_ and power output

There were no significant changes from pre- to posttest in VO_2max_ (*ES* = 0.28) or in peak power output (*ES* = 0.10) in the low cadence group. However, the freely chosen cadence group showed a significant increase in VO_2max_ from pre- to posttest (*ES* = 1.17), but not in peak power output (ES = 0.10) (Table 1).

**Table 1:** **Maximal oxygen consumption (VO_2max_) and peak power output (mean ± *SD*) pre and post training intervention period for the low cadence training group (Lctg) and the freely chosen cadence group (Fccg)**.

	**Lctg (*n* = 11)**			**Fccg (*n* = 11)**		
	**Pre**	**Post**	**Difference**	**Pre**	**Post**	**Difference**
VO_2max_ (ml · kg^−1^ · min^−1^)	57.2 ± 4.6	58.5 ± 4.8	1.4 ± 2.4	58.9 ± 2.4	62.2 ± 3.2^*^	3.3 ± 2.8
Peak power output (W)	393 ± 35	390 ± 27	−3.5 ± 19	412 ± 61	406 ± 59	−6.1 ± 13

*VO_2max_ and peak power output are average values obtained during the final minute of the test*.

* *p < 0.05 (paired sample t-test, within-group analyses from pre- to posttest)*.

A similar pattern was seen at the lactate threshold test. No significant changes were seen in the low cadence training group from pre- to post (*ES* = 0.42 for VO_2_ consumption, and 0.30 for power output), whereas the freely chosen cadence group showed significantly increased VO_2_ consumption (*ES* = 0.66) and power output at lactate threshold (*ES* = 0.21) (Table 2).

**Table 2:** **Oxygen consumption (VO_2_) and power output (mean ± *SD*) at the lactate thresholds (⊖L) (OBLA 4 mmol/L) pre and post training intervention period for the low cadence training group (Lctg) and the freely chosen cadence group (Fccg)**.

	**Lctg (*n* = 11)**			**Fccg (*n* = 11)**		
	**Pre**	**Post**	**Difference**	**Pre**	**Post**	**Difference**
VO_2_ (ml · kg^−1^ · min^−1^)	47.8 ± 3.0	49.2 ± 3.6	1.5 ± 2.8	49.4 ± 3.8	51.8 ± 3.5^*^	2.4 ± 2.2
Power output at ⊖L (W)	273 ± 30	283 ± 36	10 ± 16	284 ± 47	294 ± 48^*^	10 ± 10
% of VO_2max_	83.8 ± 5.1	84.3 ± 3.9	0.5 ± 5.7	83.9 ± 4.7	83.5 ± 4.6	−0.5 ± 5.5

* *p < 0.05 (paired sample t-test, within-group analyses from pre- to posttest)*.

There was no significant change in freely chosen cadence (*ES* = 0.13 for the low cadence group, and 0.14 for the freely chosen cadence group) or in gross efficiency (*ES* = 0.21 and 0.61) within groups on the lactate threshold test (Table 3).

**Table 3:** **Freely chosen cadence (FCC) and gross efficiency (GE) (mean ± *SD*) during the lactate threshold test and during the 30 min performance test pre and post training intervention period for the low cadence training group (Lctg) and the freely chosen cadence group (Fccg)**.

	**Lctg (*n* = 11)**			**Fccg (*n* = 11)**		
	**Pre**	**Post**	**Difference**	**Pre**	**Post**	**Difference**
**LACTATE THRESHOLD TEST**						
FCC (rpm^a^)	95 ± 9	94 ± 6	−0.8 ± 7.3	94 ± 7	95 ± 7	0.5 ± 5.1
GE	20.5 ± 1.5	20.2 ± 1.3	−0.3 ± 1.6	20.6 ± 0.6	20.2 ± 0.7	−0.4 ± 0.6
**30 MINUTES PERFORMANCE TEST**						
FCC (rpm^a^)	100 ± 6	102 ± 6	2.4 ± 5.0^*^	101 ± 7	98 ± 8	−2.7 ± 6.2^*^
GE	20.0 ± 0.6	19.8 ± 0.7	−0.2 ± 0.9	20.1 ± 1.2	20.2 ± 1.1	0.1 ± 0.8

a *revolutions per minute*.

* *p < 0.05 (independent sample t-test for the difference in the change between groups)*.

### The cycling performance test

The low cadence group showed no changes from pre to post on the 30 min performance test (*ES* = 0.02 for VO_2_ consumption, and 0.07 for mean power output), whereas the freely chosen cadence group showed a significant increase in work capacity (increased VO_2_ and mean power output) from pre- to posttest (*ES* = 0.58 and 0.28, respectively) (Table 4).

**Table 4:** **Oxygen consumption (VO_2_) (mean ± *SD*) during the 30 min performance test pre and post training intervention period for the low cadence training group (Lctg) and the freely chosen cadence group (Fccg)**.

	**Lctg (*n* = 11)**			**Fccg (*n* = 11)**		
	**Pre**	**Post**	**Difference**	**Pre**	**Post**	**Difference**
VO_2_ (ml · kg^−1^ · min^−1^)	51.6 ± 3.8	51.5 ± 4.9	−0.1 ± 2.6	52.8 ± 3.0	54.7 ± 3.5^*^	1.9 ± 2.2
Mean power output (W)	278 ± 23	276 ± 35	−3 ± 18	284 ± 42	297 ± 50^*^	13 ± 16
% of VO_2max_	90.5 ± 5.7	88.0 ± 3.7	−2.5 ± 6.8	89.7 ± 4.2	88.0 ± 3.4	−1.7 ± 3.7

*VO_2_ was measured continuously during three 2-min periods (from 8–10, 18–20, and 28–30), and an average of those periods are presented. Mean power output (W) are presented as an average of the entire test (0–30 min)*.

* *p < 0.05 (paired sample t-test, within-group analyses from pre- to posttest)*.

There was no significant change in freely chosen cadence within groups (*ES* = 0.33 for the low cadence group, and 0.40 for the freely chosen cadence group). However, a significant difference was seen when comparing the change in freely chosen cadence from pre- to posttest between the low cadence training group (2.4 ± 5.0) and the freely chosen cadence training group (−2.7 ± 6.2). There was found no significant changes on gross efficiency for neither of the two groups from pre- to posttest (*ES* = 0.31 for the low cadence group, and 0.09 for freely chosen cadence group) (Table 3).

### Leg strength

There was no significant effect on leg strength (kg) neither from pre- to posttest for the low cadence group (*ES* = 0.03, 0.05, and 0.18, 0.27, for leg press and leg extension respectively) (*n* = 7) nor for the freely chosen cadence group (*ES* = 0.12, 0.08, and 0.13, 0.14) (*n* = 9) (Table 5).

**Table 5:** **Leg strength (mean ± *SD*) pre and post training intervention period for the low cadence training group (Lctg) and the freely chosen cadence group (Fccg)**.

	**Lctg (*n* = 7)**			**Fccg (*n* = 9)**		
	**Pre**	**Post**	**Difference**	**Pre**	**Post**	**Difference**
ONE LEG PRESS (1 RM^*^ IN kg)						
Right	151 ± 31	152 ± 34	1 ± 4	128 ± 14	130 ± 18	2 ± 8
Left	150 ± 38	148 ± 37	−1 ± 5	122 ± 12	123 ± 14	1 ± 7
ONE LEG EXTENSION (1 RM^*^ IN kg)						
Right	47 ± 8	46 ± 7	−1 ± 2	44 ± 6	45 ± 8	1 ± 3
Left	45 ± 8	44 ± 9	0 ± 3	41 ± 7	43 ± 8	2 ± 3

* *RM, repetition maximum*.

## Discussion

In the present study we investigated the effects of low cadence interval training (40 rpm) at moderate intensity (73–82% of HR_max_). For the low cadence training group our results showed no significant changes in any of the aerobic variables or in power output, gross efficiency, cadence or in leg strength after the 12 weeks of low cadence training. By contrast the freely chosen cadence group, that did moderate training at freely chosen cadence, showed significant increases in VO_2max_, in VO_2_ at lactate threshold and during the 30 min cycling performance test. In addition they showed significant increase in power output at lactate threshold and in mean power output during the 30 min performance test. A significant difference was in addition seen when comparing the change in freely chosen cadence during the 30 min performance test from pre- to post between the groups.

The current study's finding is in contrast to previous studies that have demonstrated positive effects of low cadence training (Paton et al., 2009; Nimmerichter et al., 2012). In the study by Nimmerichter et al. (2012) the training was performed at higher intensity (85–90% of HR_max_ vs. 73–82% of HR_max_), and with higher cadence (60 rpm vs. 40 rpm) than in the present study. Optimal cadence of professional road cycling is reported to be lower (71 ± 1.4 rpm) during uphill cycling than during flat terrain cycling (89.3 ± 1.0 and 92.4 ± 1 rpm) (Lucia et al., 2001), thus the study by Nimmerichter et al. (2012) used a cadence that was close to the optimal cadence for uphill cycling. Therefore, a cadence of 40 rpm, as used in the present study, might have been too low to fulfill the training principle of specificity, meaning that the transfer value to ordinary cycling is too low. Indeed, interval training at high intensity as used in the study by Nimmerichter and colleagues may have been more optimal than our low cadence training to improve aerobic capacity. To strengthen this point of view, Laursen and Jenkins (2002) found that high intensity training, in many forms, can elicit significant improvements in endurance performance in already highly trained athletes. Moreover, two sessions of low cadence interval training a week may not be the optimal number of sessions for performance improvement.

Our findings demonstrate that none of the groups had any change in maximal leg strength after the training intervention, although one of the arguments for low cadence training is improved muscle strength. No change in maximal leg strength after low cadence training may be due to differences in power and strength output required during low cadence training and when producing a 1RM effort. Low cadence training produce moderate power output (moderate strength effort and velocity), whereas measurement of 1 RM required maximal strength. On the other hand, we cannot exclude improvement in muscular endurance after low cadence training although there was no improvement in maximal leg strength.

Our results showed that the freely chosen cadence group had significant improvements on aerobic variables as well as in mean power output and in power output at lactate threshold. This may be due to improvements of peripheral cardiovascular factors (Bassett and Howley, 2000). Indeed, the freely chosen cadence group compared to participants in the low cadence group may have fulfilled more optimally the training principle of specificity due to cadence. Thus the freely chosen cadence training might be more efficient to generate cardiovascular adaptation than the low cadence training. We cannot exclude a difference in % of HRmax between the two group within zone 2 (73–82% of HR_max_) during the intervention period, meaning that the freely chose cadence group may have performed higher intensity training than the low cadence training group as the intensity zones we used are relatively wide (mean HR_max_ was 180, thus zone 2 includes a HR of 131–148).

Our result showed a significant difference in change in freely chosen cadence between groups from pre to post during the 30 min performance test. The low cadence training group increased the freely chosen cadence from 100–102 rpm, whereas the freely chosen cadence group decreased the freely chosen cadence from 101–98 rpm. This difference is interesting, and more research is needed.

Although there was no significant differences between pre- and posttest for the low cadence training group, ES measurements indicated a small difference in VO_2max_, in VO_2_, power output and gross efficiency at lactate threshold, and in freely chosen cadence and in gross efficiency in the 30 min performance. Also, in the freely chosen cadence group, ES measurements showed a small difference between pre- and posttest in gross efficiency at lactate threshold, and a medium difference in freely chosen cadence during the 30 min performance test, although the dependent *t*-test showed no significant differences.

One of the strengths of the present study is that we had a homogeneous group of participants regarding aerobic capacity, cycling performance, age, and body mass. In addition, participants were randomized into either the low cadence training group or the freely chosen cadence group, which minimize potential selection biases. Compared to other comparable studies, we also have a longer intervention period, and a higher number of participants. It would have been interesting to include an additional freely chosen cadence group which only continued their usual training between the pre- and posttest. However, it was not possible to recruit enough participants who fulfilled the relatively strict inclusion criteria.

One limitation may be that we have no information about previous training history for the participants. However, the training diary during the intervention period showed no significant difference in intensity or in weekly training hours between the two groups.

Although low cadence training did not improve cycling performance in veteran cyclists, it is possible that low cadence training may be beneficial for younger, elite cyclists due to age-related adaptation differences e.g., as beneficial technique changes (i.e., improved roundness of the stroke, which is suggested to be improved after low cadence training). Moreover, physiological adaptations may also be slower in veterans than in younger and not possible to measure after 12 weeks.

To conclude, low cadence training (40 rpm at 73–82% of HR_max_) twice a week during 12 weeks did not increase aerobic capacity, cycling performance or leg strength in highly trained veteran cyclists. On the other hand, improvement in both aerobic capacity and cycling performance was seen after freely chosen cadence training at moderate intensity, and seems to be preferable compared to low cadence training.

### Conflict of interest statement

The authors declare that the research was conducted in the absence of any commercial or financial relationships that could be construed as a potential conflict of interest.
